# (*E*)-4-Bromo-2-[(phenyl­imino)­meth­yl]phenol: a new poly­morph and thermochromism

**DOI:** 10.1107/S2053229620011560

**Published:** 2020-10-08

**Authors:** Helen E. Mason, Judith A. K. Howard, Hazel A. Sparkes

**Affiliations:** a Department of Chemistry, Durham University, South Road, Durham DH1 3LE, England; b School of Chemistry, University of Bristol, Cantock’s Close, Bristol BS8 1TS, England

**Keywords:** phenol, crystal structure, poly­morph, thermochromism

## Abstract

A new poly­morph of (*E*)-4-bromo-2-[(phenyl­imino)­meth­yl]phenol, C_13_H_10_BrNO, is reported, together with a low-temperature structure determination of the previously published poly­morph. Both poly­morphs were found to have an intra­molecular O—H⋯N hydrogen bond between the phenol OH group and the imine N atom, forming an *S*(6) ring. The crystals were observed to have different colours at room temperature, with the previously published poly­morph being more orange and the new poly­morph more yellow. The planarity of the mol­ecule in the two poly­morphs was found to be significantly different, with dihedral angles (Φ) between the two aromatic rings for the previously published ‘orange’ poly­morph of Φ = 1.8 (2)° at 120 K, while the new ‘yellow’ poly­morph had Φ = 45.6 (1)° at 150 K. It was also observed that both poly­morphs displayed some degree of thermochromism and upon cooling the ‘orange’ poly­morph became more yellow, while the ‘yellow’ poly­morph became paler upon cooling.

## Abstract 2

A novel poly­morph of (*E*)-4-bromo-2-[(phenyl­imino)­meth­yl]phenol is reported, with a dihedral angle between the planes of the two aromatic rings of 45.6 (1)°, significantly different to that of the previously published poly­morph. The structure contains an intra­molecular O—H⋯N hydrogen bond forming an *S*(6) ring.

## Introduction   

A wide range of *N*-salicylideneanilines, Schiff bases of salicyl­aldehyde derivatives with aniline derivatives, have been synthesized (Özek *et al.*, 2007 ▸; Johmoto *et al.*, 2012 ▸). The *N*-salicylideneaniline derivatives are inter­esting as they have generally been found to display thermochromism, with some also showing photochromism in the solid state (Cohen & Schmidt, 1962 ▸; Cohen *et al.*, 1964 ▸; Fujiwara *et al.*, 2004 ▸). The mechanism for the chromic colour change is believed to be due to a keto–enol tautomerism (Hadjoudis & Mavridis, 2004 ▸; Robert *et al.*, 2009 ▸). The keto form is coloured, while the enol form is colourless and the switch can be induced either by changes in temperature or by irradiation. A link has been proposed between the thermochromic behaviour of a com­pound and the dihedral angle (Φ) between the two aromatic rings, with those having Φ < 25° being more likely to be strongly thermochromic (Hadjoudis & Mavridis, 2004 ▸; Robert *et al.*, 2009 ▸). A larger *inter*planar angle allows increased orbital overlap and greater delocalization into the π-system, which reduces the basicity of the N atom and thus the thermochromism. The effect of substituents on the OH bond strength, nitro­gen-accepting ability and crystal packing have also been postulated as important in the chromic behaviour of the *N*-salicylideneanilines (Hadjoudis & Mavridis, 2004 ▸; Robert *et al.*, 2009 ▸). It has also been observed that, in general, the *N*-salicylideneanilines that are more strongly coloured, typically red/orange, at room temperature, tend to be more strongly thermochromic than those that are paler, typically yellow, at room temperature (Ogawa *et al.*, 2001 ▸; Fujiwara *et al.*, 2009 ▸).

The structures of (*E*)-4-halogeno-2-[(phenyl­imino)­meth­yl]phenol have been reported for fluoro (Swetha *et al.*, 2017 ▸), chloro (Bregman *et al.*, 1964 ▸; Ogawa *et al.*, 1998 ▸), bromo (Yan *et al.*, 2014 ▸) and iodo (Swetha *et al.*, 2019 ▸). Herein a new poly­morph of (*E*)-4-bromo-2-[(phenyl­imino)­meth­yl]phenol, denoted **1B**, is reported together with a new low-temperature determination of the previously reported poly­morph, **1A** (Yan *et al.*, 2014 ▸). Both poly­morphs were found to be thermochromic to some extent.
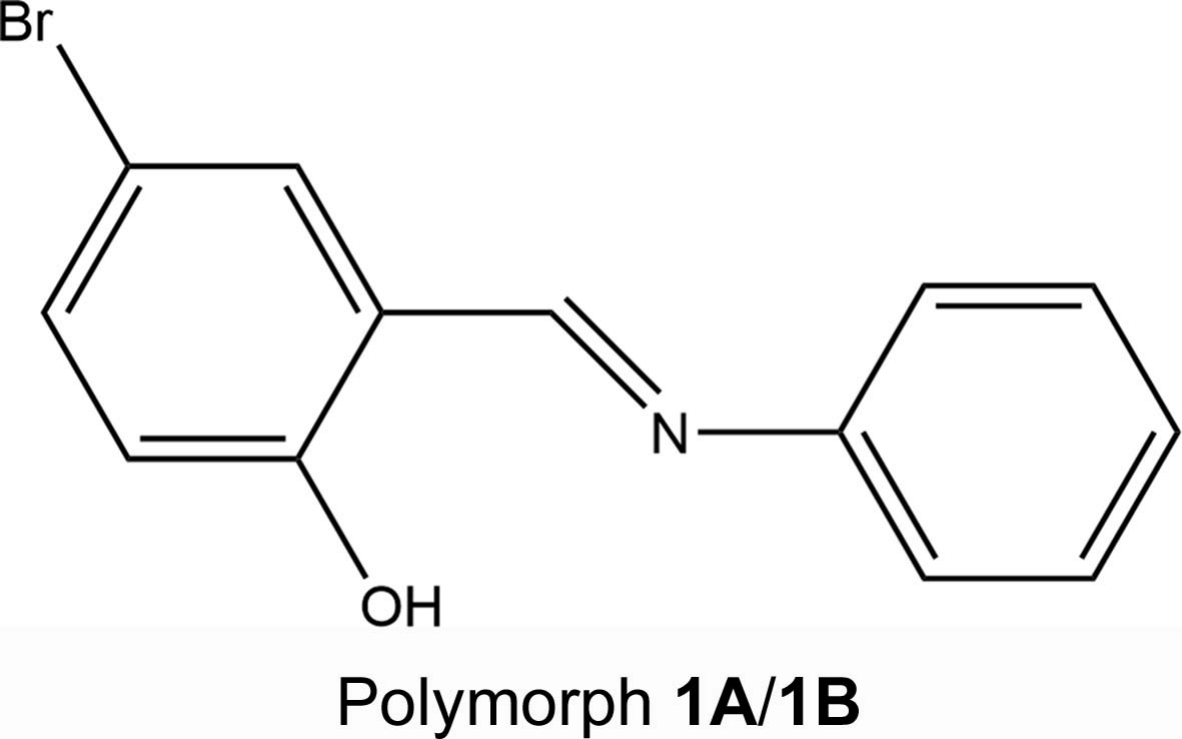


## Experimental   

### Synthesis and crystallization   

(*E*)-4-Bromo-2-[(phenyl­imino)­meth­yl]phenol was synthesized by direct condensation of 5-bromo­salicyl­aldehyde and aniline in ethanol. The two materials (0.005 mol of each, 1.000 g of 5-bromo­salicyl­aldehyde and 0.466 g of aniline) were dissolved separately in ethanol (25 ml). The resultant solutions were combined and refluxed with stirring for 4 h. After removal of any precipitate, the solution was rotary evaporated until further precipitate formed, the solid filtered off, rinsed with ethanol and left to dry, giving a yield of 94% (1.304 g, 0.0047 mol). Yellow single crystals (of **1B**) crashed out of the crude reaction mixture and orange single crystals (of **1A**) were produced by recrystallization from ethanol.

### Refinement   

Crystal data, data collection and structure refinement details are summarized in Table 1 ▸. All H atoms, apart from the OH hydrogen involved in the intra­molecular hydrogen bonding with the imine N atom, were positioned geometrically and refined using a riding model. The H atoms involved in the intra­molecular hydrogen bond were located in the Fourier difference map wherever feasible. In **1A**, the O—H distance was restrained to 0.86 (1) Å.

## Results and discussion   

**Figure 1:**
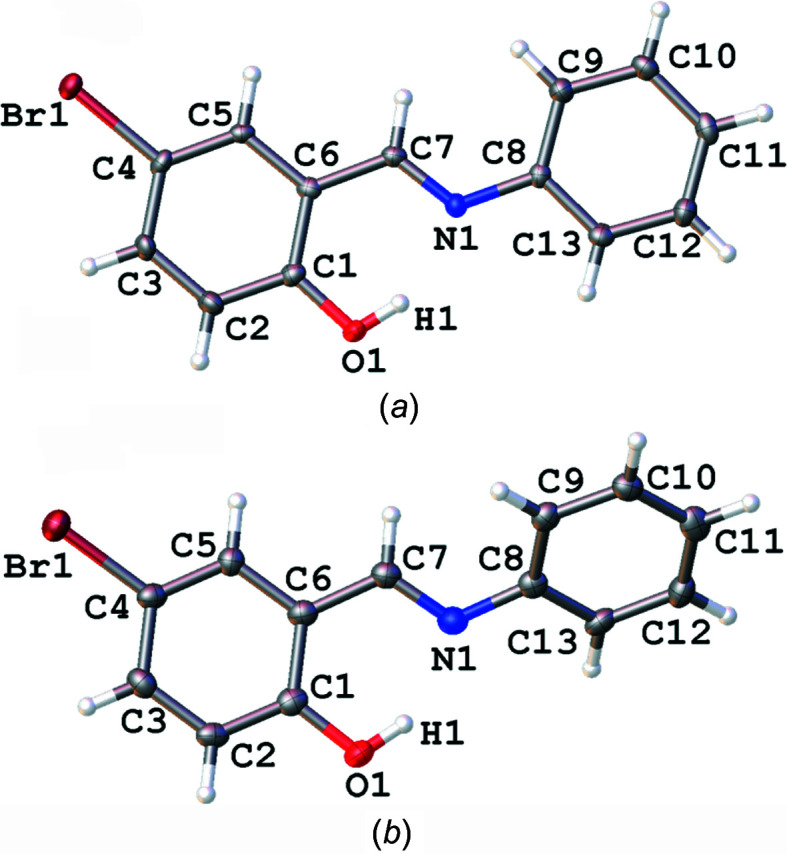
Illustration of the structures of (*a*) **1A** and (*b*) **1B** at 120 (2) and 150 (2) K, respectively, with the atomic numbering schemes depicted. Anisotropic displacement parameters are shown at the 50% probability level.

**Figure 2:**
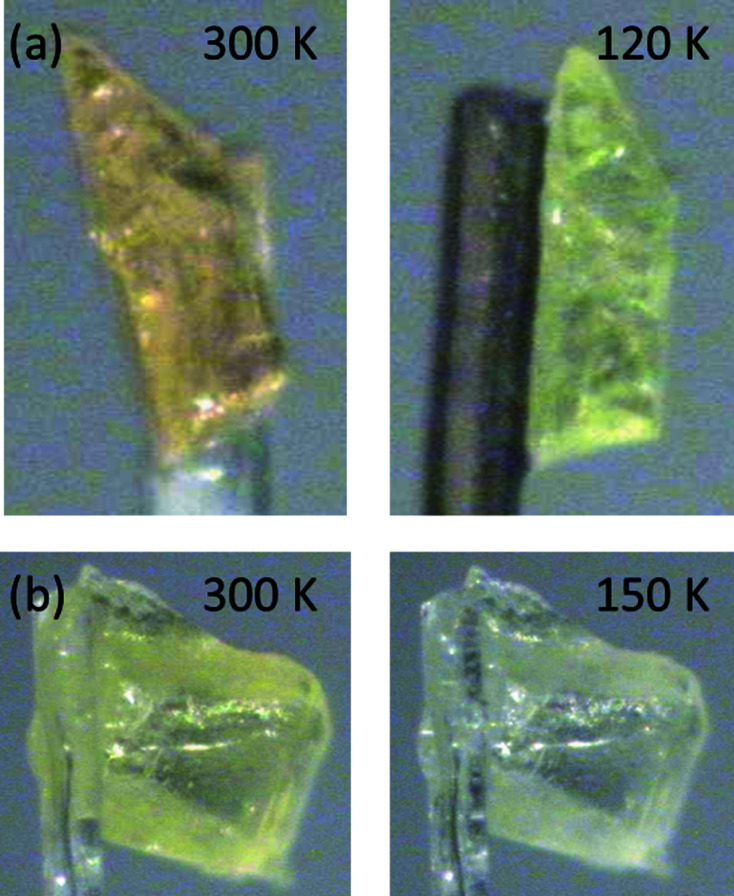
Illustration of the colour change observed upon cooling (*a*) **1A** and (*b*) **1B**.

The structures of poly­morphs **1A** and **1B** are shown in Fig. 1 ▸. The structure of **1A** at 120 K was consistent with the previously published structure at room temperature (Yan *et al.*, 2014 ▸). The structure of **1A** was obtained in the ortho­rhom­bic space group *Pca*2_1_, while **1B** was obtained in the monoclinic space group *Cc*. The com­pound consists of a hy­droxy-substituted phenyl ring linked *via* an imine group to a second unsubstituted phenyl group. In both poly­morphs, the structures were found to exist in the enol form, with C7=N1 bond lengths of 1.282 (4) Å for **1A** and 1.284 (10) Å for **1B**, indicating a double bond, and C1—O1 bond lengths of 1.350 (5) Å for **1A** and 1.351 (9) Å for **1B**, indicating a single bond. The structures showed quite different dihedral angles, with **1A** having Φ = 1.8 (2)° at 120 K and **1B** having Φ = 45.6 (1)° at 150 K. Upon cooling, the structures were both found to display some degree of thermochromism with **1A** changing from orange at room temperature to yellow at 120 K and **1B**, which was yellow at room temperature, becoming slightly paler at 150 K (Fig. 2 ▸). The differences in the thermo­chromic behaviour of the two poly­morphs are consistent with literature suggestions that a larger dihedral angle increases the overlap of the π-system reducing the nitro­gen basicity, disfavouring the keto form and thus also reducing the thermochromism of the com­pound.

**Figure 3:**
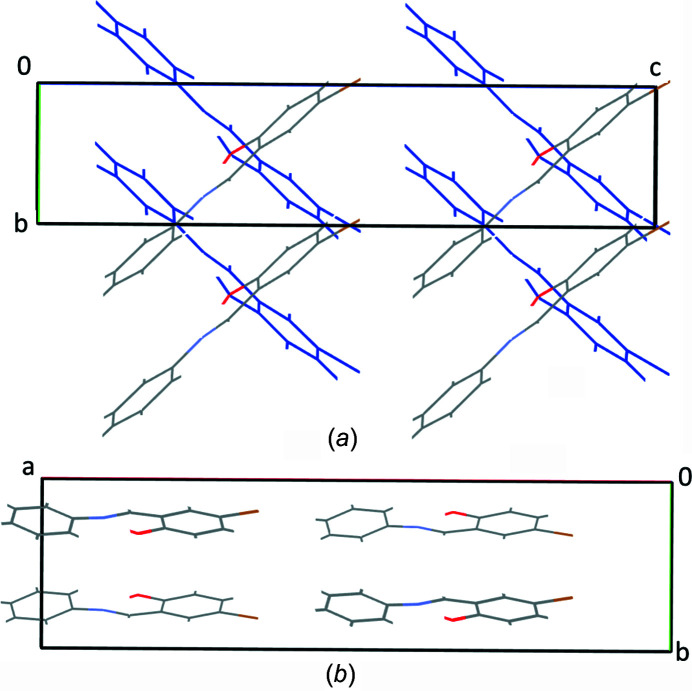
(*a*) Illustration of the packing for **1A**, looking down the *a* axis; mol­ecules in blue are in-plane behind those in element colours. (*b*) View of polymorph **1B**, looking down the *c* axis.

**Figure 4:**
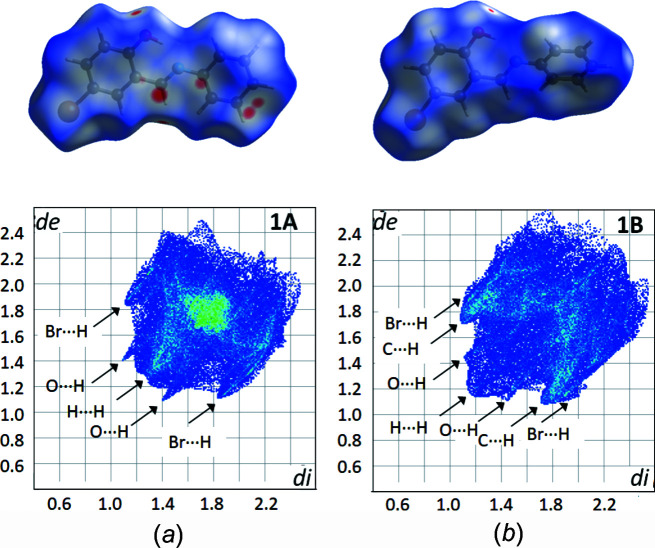
The Hirshfeld surface plot (top) and fingerprint plot (bottom) for (*a*) **1A** and (*b*) **1B**.

An intra­molecular O1—H1⋯N1 hydrogen bond, involving the phenol OH group and imine N atom, was identified in the structures of both poly­morphs and creates an *S*(6) ring. The hydrogen-bonding parameters were almost identical in the two structures, with a donor–acceptor distance of ∼2.59 Å and a hydrogen-bond angle of ∼150° (Tables 2 ▸ and 3 ▸.). The packing of the two poly­morphs was unsurprisingly significantly different given the large difference in the dihedral angles. In poly­morph **1A**, the mol­ecules are essentially planar and orientated diagonally such that the plane of the mol­ecule is perpendicular to the *bc* plane and, as a result of the 2_1_ screw axis, the diagonal slant of alternate mol­ecules along the *a*-axis direction essentially align in opposite directions (Fig. 3 ▸ *a*). It was also noted that there were short π-type contacts between the C=N group and the phenol ring in the 0
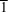
1 direction, with a centroid-to-centroid (C=N) distance of 3.326 (1) Å. These can be seen on the Hirshfeld surface of **1A** as red dots (Fig. 4 ▸ *a*). In poly­morph **1B**, although the mol­ecules themselves are twisted, the mol­ecules are orientated relative to each other such that they create planes parallel to the *ac* plane direction (see Fig. 3 ▸ *b*).

Examining the Hirshfeld fingerprint plots (Turner *et al.*, 2017 ▸) for the two structures highlights the differences in the two structures, not least in the shapes of the two plots (Fig. 4 ▸). For **1A**, the O⋯H and Br⋯H contacts are quite obvious, while in **1B** the H⋯H and C⋯H contacts are significantly more pronounced, slightly masking the O⋯H and Br⋯H contacts. These differences are very apparent on the Hirshfeld surface for both com­pounds with a greater number of red spots on the surface of **1A** that are more noticeable than for **1B**, showing that **1A** has more short contacts.

The two poly­morphs of (*E*)-4-bromo-2-[(phenyl­imino)­meth­yl]phenol reported herein are particularly inter­esting as part of a study into *N*-salicylideneanilines because they show significantly different mol­ecular conformations and colours at room temperature. In line with the literature, the extent of the thermochromism was found to be linked to the dihedral angle, with **1A** [Φ = 1.8 (2)°] showing a greater colour change upon cooling than observed for **1B** [Φ = 45.6 (1)°].

## Supplementary Material

Crystal structure: contains datablock(s) 1A, 1B, global. DOI: 10.1107/S2053229620011560/yd3009sup1.cif

Structure factors: contains datablock(s) 1A. DOI: 10.1107/S2053229620011560/yd30091Asup2.hkl

Structure factors: contains datablock(s) 1B. DOI: 10.1107/S2053229620011560/yd30091Bsup3.hkl

CCDC references: 2025079, 2025078

## Figures and Tables

**Table 1:** Experimental details For both structures: C_13_H_10_BrNO, *M*_r_ = 276.13, *Z* = 4. Experiments were carried out with Mo *K*α radiation using an Oxford Diffraction Xcalibur (Sapphire3, Gemini ultra) diffractometer. An analytical absorption correction [*CrysAlis PRO* (Oxford Diffraction, 2010 ▸), based on expressions derived by Clark & Reid (1995 ▸)] was used. Refinement was with 2 restraints. H atoms were treated by a mixture of independent and constrained refinement.

	Polymorph **1A**	Polymorph **1B**
Crystal data		
Crystal system, space group	Orthorhombic, *P* *c* *a*2_1_	Monoclinic, *C* *c*
Temperature (K)	120	150
*a*, *b*, *c* (Å)	12.2768 (3), 4.4829 (1), 19.6694 (4)	25.8944 (13), 6.9439 (4), 6.1499 (4)
α, β, γ (°)	90, 90, 90	90, 91.381 (5), 90
*V* (Å^3^)	1082.52 (4)	1105.48 (11)
μ (mm^−1^)	3.77	3.69
Crystal size (mm)	0.46 × 0.20 × 0.05	0.58 × 0.49 × 0.22

Data collection		
*T*_min_, *T*_max_	0.383, 0.847	0.190, 0.585
No. of measured, independent and observed [*I* > 2σ(*I*)] reflections	13133, 2215, 2133	7049, 2254, 2142
*R*_int_	0.043	0.051
(sin θ/λ)_max_ (Å^−1^)	0.625	0.625

Refinement		
*R*[*F* ^2^ > 2σ(*F* ^2^)], *wR*(*F* ^2^), *S*	0.022, 0.053, 1.05	0.039, 0.100, 1.05
No. of reflections	2215	2254
No. of parameters	149	148
Δρ_max_, Δρ_min_ (e Å^−3^)	0.39, −0.23	0.95, −0.34
Absolute structure	Flack *x* determined using 993 quotients [(*I* ^+^) − (*I* ^−^)]/[(*I* ^+^) + (*I* ^−^)] (Parsons *et al.*, 2013 ▸)	Flack *x* determined using 1007 quotients [(*I* ^+^) − (*I* ^−^)]/[(*I* ^+^) + (*I* ^−^)] (Parsons *et al.*, 2013 ▸)
Absolute structure parameter	−0.006 (8)	−0.010 (19)

Computer programs: *CrysAlis PRO* (Oxford Diffraction, 2010 ▸), *SHELXS97* (Sheldrick, 2008 ▸), *SHELXL2018* (Sheldrick, 2015 ▸) and *OLEX* (Dolomanov *et al.*, 2009 ▸).

**Table 2:** Hydrogen-bond geometry (Å, °) for poly­morph **1A** [Chem scheme1]

*D*—H⋯*A*	*D*—H	H⋯*A*	*D*⋯*A*	*D*—H⋯*A*
O1—H1⋯N1	0.86 (1)	1.82 (3)	2.593 (4)	150 (5)

**Table 3:** Hydrogen-bond geometry (Å, °) for poly­morph **1B** [Chem scheme1]

*D*—H⋯*A*	*D*—H	H⋯*A*	*D*⋯*A*	*D*—H⋯*A*
O1—H1⋯N1	0.86 (11)	1.82 (11)	2.590 (10)	148 (10)
